# High genetic diversity in hard ticks from a China-Myanmar border county

**DOI:** 10.1186/s13071-018-3048-5

**Published:** 2018-08-14

**Authors:** Lan-Hua Li, Yi Zhang, Jia-Zhi Wang, Xi-Shang Li, Shou-Qin Yin, Dan Zhu, Jing-Bo Xue, Sheng-Guo Li

**Affiliations:** 1 Health Shandong Collaborative Innovation Center for Major Social Risk Prediction and Management, School of Public Health and Management, Weifang Medical University, Weifang, 261053 People’s Republic of China; 2 National Institute of Parasitic Diseases, Chinese Center for Disease Control and Prevention, Shanghai, 200025 People’s Republic of China; 3 Tengchong Center for Disease Control and Prevention, Tengchong, 679100 People’s Republic of China

**Keywords:** Hard tick, Genetic diversity, *16S* rRNA gene, *cox*1 gene, Tengchong County, China

## Abstract

**Background:**

Many tick species have great morphological similarity and are thus grouped into species complexes. Molecular methods are therefore useful in the classification and identification of ticks. However, little is known about the genetic diversity of hard ticks in China, especially at the subspecies level. Tengchong is one of the epidemic foci of tick-borne diseases in China, but the tick species inhabiting the local area are still unknown.

**Methods:**

Eighteen villages in Tengchong County, China, were selected for sampling carried out from September to October 2014. Infesting hard ticks were removed from the body surface of domestic animals and questing ticks were collected from grazing fields. After morphological identification, molecular characteristics of each tick species were analyzed based on both *16S* rRNA and cytochrome *c* oxidase subunit 1 (*cox*1) gene fragments.

**Results:**

Six tick species were identified based on morphology: *Rhipicephalus microplus*, *R. haemaphysaloides*, *Ixodes ovatus*, *Haemaphysalis longicornis*, *H. shimoga* and *H. kitaokai*. Phylogenetic analysis using the *cox*1 gene revealed that *R. microplus* ticks from the present study belong to clade C. For tick samples of both *R. haemaphysaloides* and *I. ovatus*, three phylogenetic groups were recognized, and the intergroup genetic distances exceeded the usual tick species boundaries. *Haemaphysalis longicornis* ticks were clustered into two separate clades based on the *cox*1 gene. For ticks from both *H. shimoga* and *H. kitaokai*, two phylogenetic groups were recognized based on the phylogenetic analysis of the *16S* rRNA gene, and the intergroup genetic distances also exceeded the known boundaries for closely related tick species.

**Conclusions:**

According to molecular analyses, new species or subspecies closely related to *R. haemaphysaloides*, *I. ovatus*, *H. shimoga* and *H. kitaokai* probably exist in the China-Myanmar border Tengchong County, or these ticks form species complexes with highly divergent mitochondrial lineages. Morphological comparisons are warranted to further confirm the taxonomic status of these tick species.

**Electronic supplementary material:**

The online version of this article (10.1186/s13071-018-3048-5) contains supplementary material, which is available to authorized users.

## Background

Hard ticks (Acari: Ixodidae) are obligate blood-sucking parasitic arthropods which can infest reptiles, birds and mammals [1]. They transmit a wide range of pathogens of both veterinary and medical importance, including bacteria, viruses, protozoa, etc. Moreover, new pathogens are continuously being identified from ticks [2, 3]. Therefore, hard ticks are usually considered to be the most important vectors of pathogens in the temperate zone [4].

China covers approximately 9.6 million square kilometers of land area. At least 117 tick species from seven genera have been reported in China [5]. In recent years, the reported number of tick-borne infections has increased in this country. Since 1982, more than 30 emerging tick-borne agents have been recognized [1, 6–9], including severe fever with thrombocytopenia syndrome virus, *Borrelia burgdorferi* (*sensu lato*), *Babesia* spp., the spotted fever group rickettsiae, *Ehrlichia*, *Anaplasma*, etc. Thus, tick-borne diseases are considered to be an emerging threat to public health in mainland China.

Identification of tick species is essential to the control of tick-borne diseases. However, traditional morphological identification requires extensive experience and can be challenging when the specimens are engorged with blood, in immature stages (larva or nymph stage) or physically damaged [10]. Therefore, molecular methods based on DNA barcoding, including the *16S* rRNA gene and the cytochrome *c* oxidase subunit 1 (*cox*1) gene, are useful in the classification of ticks. Moreover, many tick species have great morphological similarity and are thus grouped into species complexes. Thus, molecular analysis can expand our knowledge of ticks. However, little is known about the genetic diversity of hard ticks in China, especially at the subspecies level.

Tengchong County of Yunnan Province is located on the southwest border of China (24°38'–25°52'N, 98°05'–98°45'E). It is adjacent to Myanmar on the west, and more than 70% of the land is covered by forest. The county is inhabited by a wide variety of wild animals and arthropods. Several human cases of tick-borne infections have also been reported there in recent years [11]. Hence, Tengchong is considered to be one of the epidemic foci of tick-borne diseases. However, the tick species inhabiting the local area are still unknown.

In the present study, we conducted a survey from September to October 2014, to investigate the distribution of tick species in Tengchong County. Molecular classification was then performed, and genetic diversity was analyzed for both the *16S* rRNA gene and the *cox*1 mitochondrial gene.

## Methods

### Tick collection and morphological identification

**Fig. 1:**
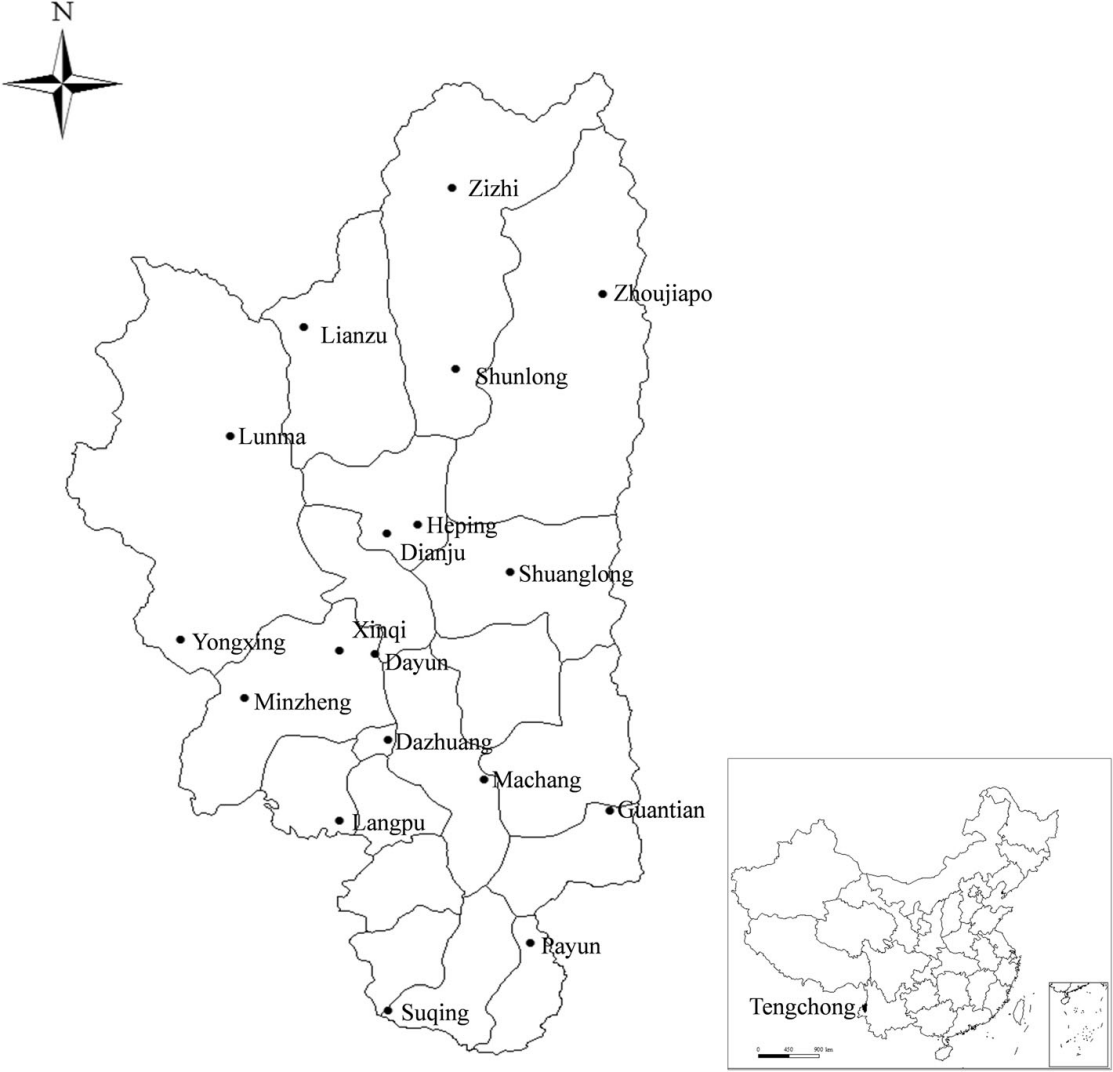
Geographical locations for tick collection in the present study

A total of 18 villages with a relatively large amount of livestock were arbitrary selected from 14 towns of Tengchong County (Fig. 1 and Table 1). Infesting ticks were collected from the body surface of domestic animals, including cattle, goats and dogs, from September to October 2014. After collection of infesting ticks, questing ticks were collected with a white drag cloth from the grazing locations. The ticks were preserved in 75% ethanol for further analysis. All ticks were identified to the species level under a dissecting microscope (Olympus Corporation, Tokyo, Japan) based on morphology [12]. The specimens were then stored at -20 °C for subsequent processing.

**Table 1:** Information on locations of tick collection and distribution of tick species in Tengchong County, Yunnan Province, China

Location^a^	Latitude	Longitude	Elevation (m)	*R. m.*	*R. h.*	*I. o.*	*H. l.*	*H. k.*	*H. s.*
Suqing, Xinhua	24.68195	98.46520	1110	×	×				
Langpu, Hehua	24.92036	98.40881	1334						
Payun, Tuantian	24.76645	98.63097	1469		×				
Shuanglong, Qushi	25.23325	98.60783	1542	×					
Dazhuang, Heshun	25.02243	98.46550	1579	×	×	×			
Minzheng, Zhonghe	25.13423	98.40763	1724	×		×			
Dianju, Gudong	25.29312	98.49935	1763	×					
Yongxing, Houqiao	25.14863	98.22210	1812	×	×			×	
Heping, Gudong	25.28138	98.46355	1812	×					
Xinqi, Zhonghe	25.07495	98.29690	1824			×			
Guantian, Wuhe	24.93287	98.72402	1830	×	×	×			
Shunlong, Mingguang	25.48820	98.54453	1857	×			×		
Lunma, Houqiao	25.40383	98.28007	1966	×				×	×
Zhoujiapo, Jietou	25.58273	98.71622	1986	×		×			
Zizhi, Mingguang	25.71568	98.54013	2059	×			×		
Lianzu, Diantan	25.54147	98.36693	2071	×		×			
Machang, Mangbang	24.97200	98.57728	2163	×		×	×		
Dayun, Mazhan	25.12965	98.44957	2560	×		×			

*Abbreviations*: *R. m*. *Rhipicephalus microplus*, *R. h. R. haemaphysaloides*, *I. o*. *Ixodes ovatus*, *H. l. Haemaphysalis longicornis*, *H. k. H. kitaokai*, *H. s. H. shimoga*

^a^Village, town

### DNA extraction

Ticks were rinsed 3 times for 1 min by vortex in 75% ethanol and then washed 3 times in sterile phosphate-buffered saline (PBS). DNA was extracted individually with a DNeasy Blood & Tissue Kit (Qiagen, Hilden, Germany) according to the manufacturer’s protocol.

### PCR amplification of the tick *16S* rRNA gene and *cox*1 gene

For each tick species, at least 15 DNA specimens were selected for PCR amplification using primers targeting the *16S* rRNA gene fragment (16S+1: 5'-CCG GTC TGA ACT CAG ATC AAG T-3' and 16S-1: 5'-CTG CTC AAT GAT TTT TTA AAT TGC TGT GG-3') [13]. For tick species with less than 15 individuals, all of the DNA samples were PCR amplified. As two *Ixodes ovatus* groups were identified by subsequent molecular analysis, all of the specimens of *I. ovatus* ticks were PCR amplified using the primer set 16S+1 and 16S-1. The PCR was performed using a C1000 Touch™ Thermal Cycler (Bio-Rad Laboratories, Irvine, CA, USA) under the following conditions: 95 °C for 5 min, followed by 35 cycles of 95 °C for 30 s, 57 °C for 30 s and 72 °C for 40 s, ending at 72 °C for 10 min. Each 20 μl PCR mixture contained 10 μl of 2× PCR Master Mix (with dyes, DBI® Bioscience, Shanghai, China), 1 μl of each primer (5 mM), 6 μl of water and 2 μl of DNA sample. The PCR products were then purified and sequenced by Sangon Biotech (Shanghai, China).

For each tick species, at least 2 specimens were selected based on the results of the molecular analysis of the *16S* rRNA gene. The selected specimens were further PCR amplified using primers targeting the *cox*1 gene (LCO1490: 5'-GGT CAA CAA ATC ATA AAG ATA TTG G-3' and HCO2198: 5'-TAA ACT TCA GGG TGA CCA AAA AAT CA-3') [14]. The PCR for the *cox*1 gene was performed under the same conditions as the amplification of the *16S* rRNA gene fragment.

### Molecular analysis of the tick *16S* rRNA gene and *cox*1 gene

Sequences of both the *16S* rRNA gene and the *cox*1 gene were compared with the available data on GenBank using Blast on the NCBI website (https://blast.ncbi.nlm.nih.gov/Blast.cgi) after trimming the low-quality sequences at both ends.

Representative *16S* rRNA or *cox*1 gene sequences of *Rhipicephalus microplus*, *R. haemaphysaloides*, *R. sanguineus* and *R. turanicus* were downloaded from GenBank for phylogenetic analysis of *Rhipicephalus* species [15]. Similarly, *16S* rRNA or *cox*1 gene sequences of *I. ovatus* and species close to *I. ovatus* were downloaded from GenBank based on the results of BLAST, including *I. vespertilionis* and *I. canisuga* [16–18]. Using the same methods, representative sequences of several *Haemaphysalis* species were also downloaded, including *H. shimoga*, *H. erinacei*, *H. cornigera*, *H. parva* and *H. kitaokai* [19–23]. For *R. microplus* and *H. longicornis*, all of the *16S* rRNA and *cox*1 sequences of ticks from China were downloaded. Multiple sequence alignments were then conducted in Clustal W2, and the neighbor-joining (NJ) method was applied to construct a phylogenetic tree with 1000 replicates using MEGA 5.1 software [24].

To further determine the intraspecies and interspecies genetic distances among *Ixodes*, *Rhipicephalus* and *Haemaphysalis* ticks, pairwise Kimura’s 2-parameter (K2P) distances were also calculated using the *16S* rRNA gene or *cox*1 gene in MEGA.

## Results

### Morphological identification of ticks

As shown in Table 2, 981 domestic animals were examined, including 837 goats, 113 cattle and 31 dogs. Among all the examined animals, 198 (20.2%) were infested by ticks. A total of 903 infesting ticks were collected from the animals. Among all the infesting ticks, 652 were identified as *R. microplus* [25], 36 as *R. haemaphysaloides* [26], 29 as *H. longicornis* [26] and 186 as *I. ovatus* [27, 28] according to the identification key for adult ticks described by Deng & Jiang [12]. *Ixodes ovatus* and *H. longicornis* were collected only from goats, *R. microplus* ticks were collected from both goats and cattle, and *R. haemaphysaloides* were found on all three animal species (Table 2).

**Table 2:** Tick species infesting different domestic animals in Tengchong County, Yunnan Province, China

Animals	*N*	No. infested (%)	Tick species			
			*R. microplus*	*R. haemaphysaloides*	*I. ovatus*	*H. longicornis*
Goats	837	149 (17.8)	258	2	186	29
Cattle	113	47 (41.6)	394	18	0	0
Dogs	31	2 (6.5)	0	16	0	0
Total	981	198 (20.2)	652	36	186	29

A total of 558 questing ticks were collected. All of the questing ticks were larvae except for one *I. ovatus* adult. Among the questing larvae, 459 were identified as *R. microplus* (after Deng & Jiang [12]; figure 352); 62 as *H. longicornis* (after Deng & Jiang [12]; figure 167); 4 as *H. shimoga* [29]; and 32 as *H. kitaokai* [30].

### Molecular identification and classification of ticks by nucleotide BLAST

Morphological identifications for *R. microplus*, *R. haemaphysaloides* and *H. longicornis* ticks were consistent with GenBank BLAST using sequences of both the *16S* rRNA and the *cox*1 genes, with sequence identity of 98–100% to sequences of the corresponding tick species (Additional file 1: Table S1 and Additional file 2: Table S2).

For the *16S* rRNA gene sequences of *I. ovatus* from the present study, the closest sequence was from an *I. ovatus* isolate collected in Yunnan, China (GenBank: KU664519.1), with a sequence similarity of 92–97% (Additional file 1: Table S1). A total of 15 specimens of *I. ovatus* were analyzed using the *cox*1 gene. According to the results of the *cox*1 gene BLAST, 2 *I. ovatus* specimens were closest to *I. canisuga* (GenBank: KY962049.1), with a sequence similarity of 83%, while 13 other *I. ovatus* ticks were closest to an *I. ovatus* isolate from Japan (GenBank: AB231670.1), with 87–88% sequence similarity (Additional file 2: Table S2).

For the *16S* rRNA gene sequences of *H. shimoga*, the closest sequence was from a *H. shimoga* isolate collected from India (GenBank: MH044717.1), with a similarity of 95%, while the sequences of *H. kitaokai* from this study were closest to the sequence of a *H. kitaokai* isolate from Japan (GenBank: AB819202.1), with a similarity of 94% (Additional file 1: Table S1). No *cox*1 sequence of *H. shimoga* was available in the GenBank database; hence the *cox*1 sequence closest to *H. shimoga* from this study was from *I. erinacei* (GenBank: KX901844.1), with a sequence similarity of 90%. The closest *cox*1 sequences to those for *H. kitaokai* generated here were from *H. concinna* (GenBank: JX573136.1), with a sequence similarity of 90% (Additional file 2: Table S2).

### Phylogenetic analyses and genetic distances for *R. microplus* and *R. haemaphysaloides*

**Fig. 2:**
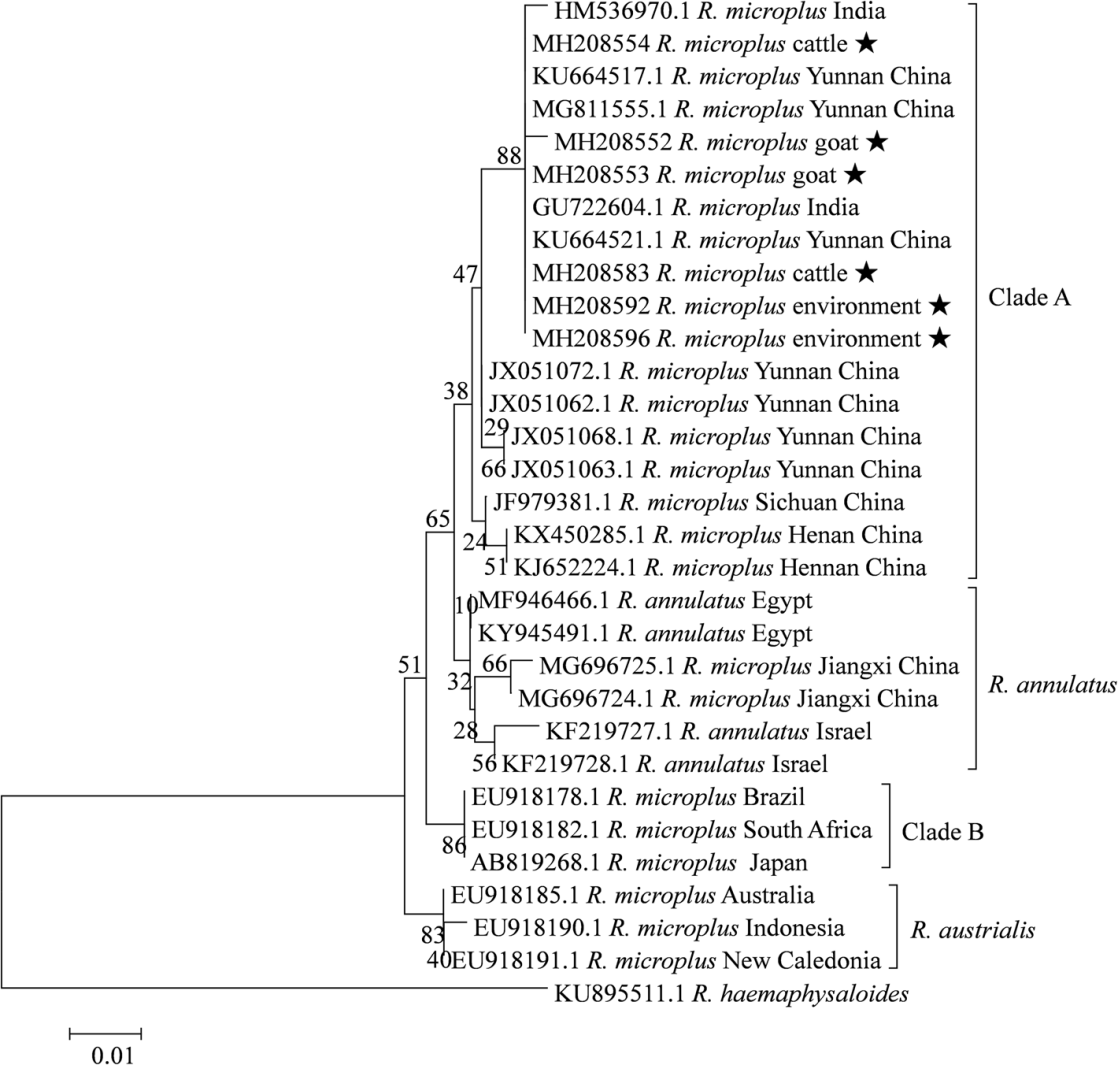
Phylogenetic tree for *R. microplus* based on the *16S* rRNA gene, including sequences obtained in the present study and representative sequences of the known subspecies-level taxa from GenBank. The sequences generated in the present study are indicated with a star

At least 4 taxa have been reported in the *R. microplus* complex based on phylogenetic analysis of the *16S* rRNA gene: *R. annulatus*, *R. australis* and *R. microplus* clades A and B [31, 32]. All *R. microplus* ticks collected during the present study were clustered into one clade, together with other *R. microplus* isolates from Yunnan Province of China and two Indian isolates (Fig. 2). The mean K2P distances within the group ranged between 0–0.008, while the distances between groups ranged between 0.010–0.022 (Additional file 3: Table S3).

According to phylogenetic analysis based on the *cox*1 gene, 5 taxa of *R. microplus* have been reported [31]. The ticks identified as *R. microplus* in the present study and two isolates from India clustered into clade B based on *cox*1 gene analysis (Additional file 4: Figure S1). The mean K2P distances within and between groups ranged between 0–0.030 and 0.059–0.100, respectively (Additional file 5: Table S4).

**Fig. 3:**
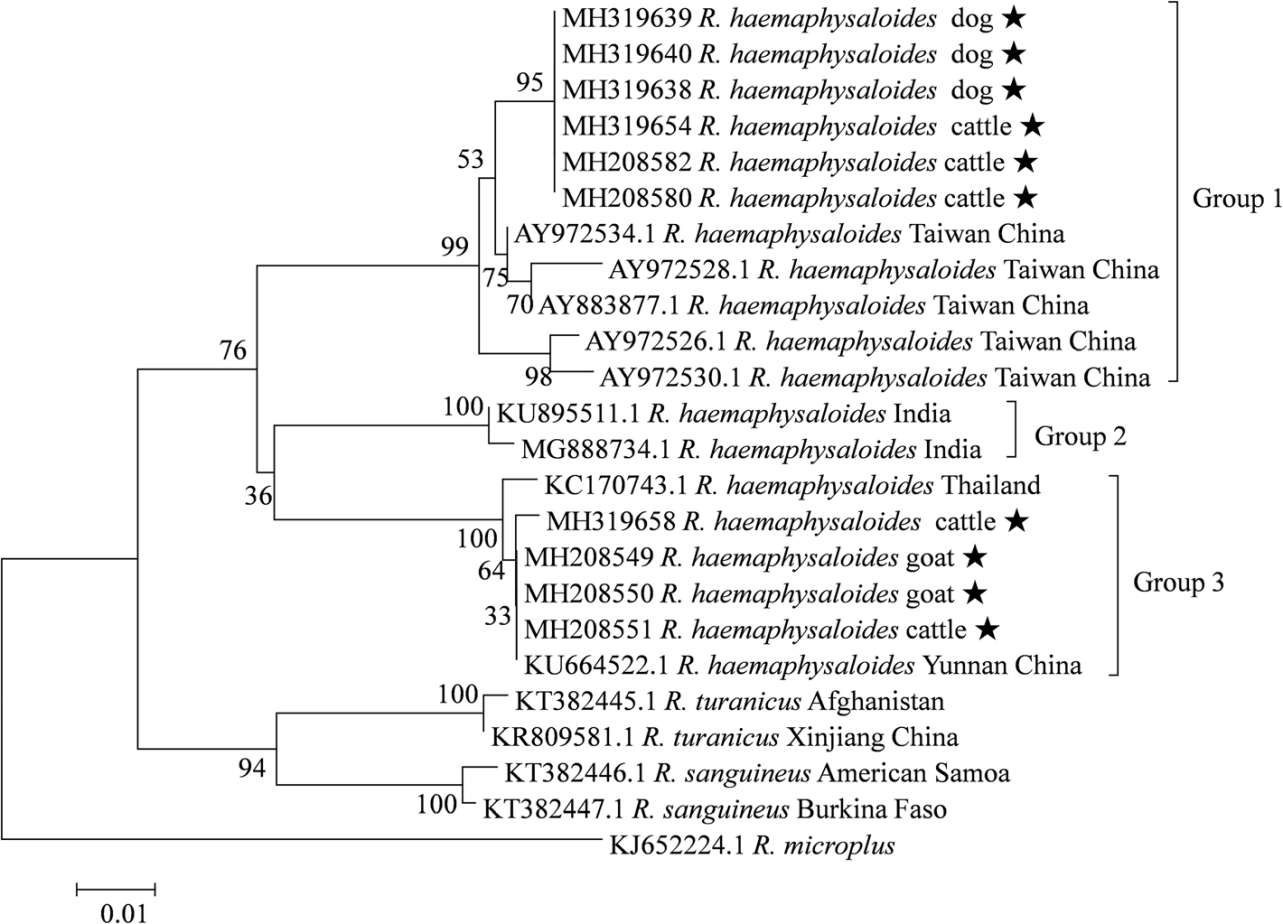
Phylogenetic tree for *R. haemaphysaloides* based on the *16S* rRNA gene, including sequences obtained in the present study and sequences retrieved from GenBank. The sequences generated in the present study are indicated with a star

For *R. haemaphysaloides* ticks, 36 sequences of *16S* rRNA gene fragments from the present study were analyzed, and 3 genetic groups were recognized. Twenty-six specimens from the present study and 10 from Taiwan of China formed Group 1; 2 ticks from India formed Group 2; and 12 specimens were clustered together and formed Group 3, including 10 from the present study, 1 from Thailand and 1 from Yunnan Province of China (Fig. 3). The mean K2P distances within groups ranged between 0–0.025, while the mean distances between groups were 0.058–0.075 (Additional file 6: Table S5).

### Phylogenetic analyses and genetic distances for *Ixodes ovatus*

**Fig. 4:**
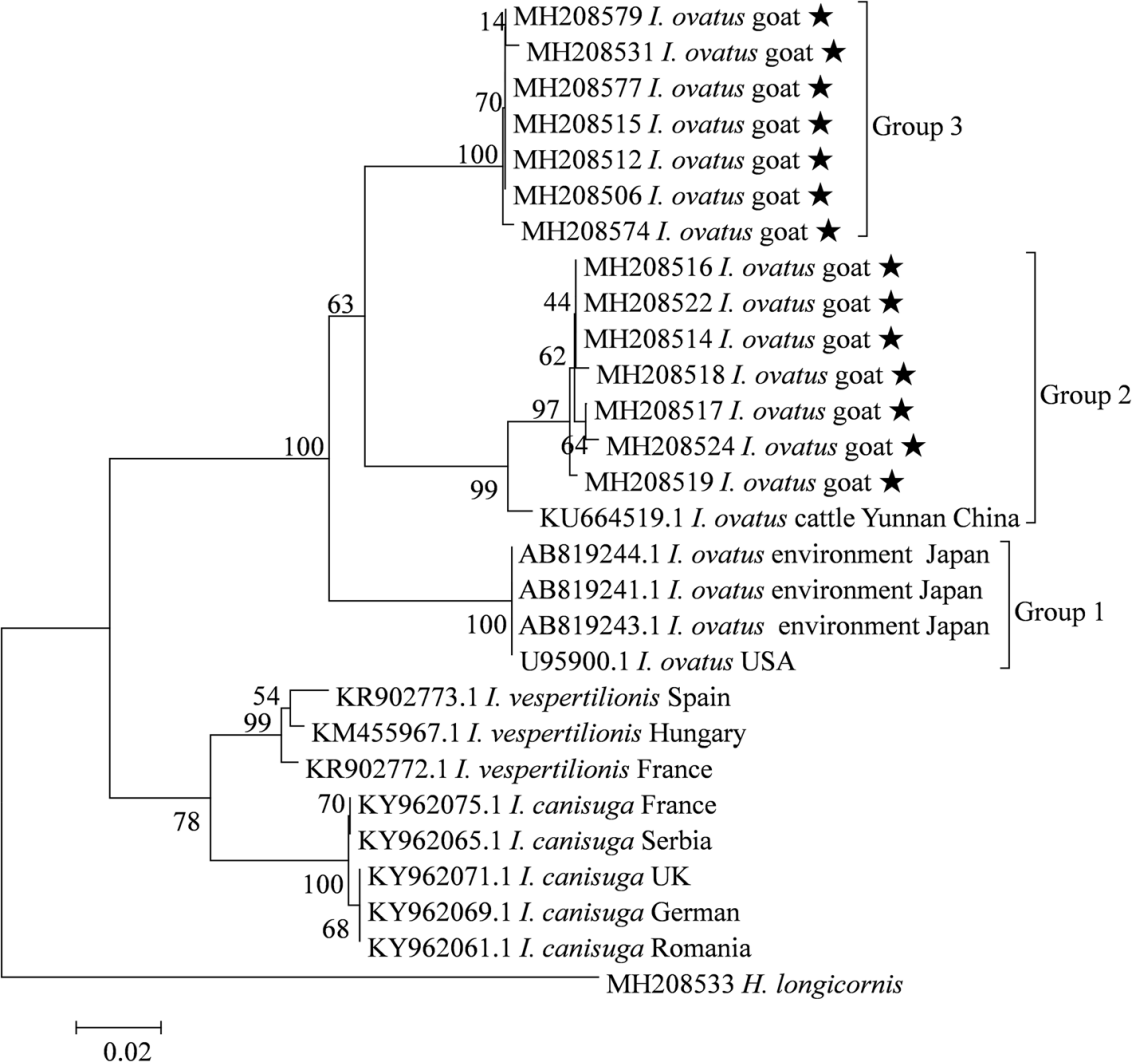
Phylogenetic tree for *I. ovatus* based on the *16S* rRNA gene, including sequences obtained in the present study and representative sequences from GenBank. The sequences generated in the present study are indicated with a star

For *I. ovatus* ticks, 34 sequences of *16S* rRNA gene from the present study were selected for phylogenetic analysis, and 3 genetic groups of *I. ovatus* were recognized. *Ixodes ovatus* collected in Japan and the USA clustered together and formed Group 1, 7 specimens from the present study and one from another location of China formed Group 2, while other specimens from the present study formed Group 3 (Fig. 4). The mean K2P distances between groups ranged between 0.079–0.102, while the mean genetic distances within groups ranged between 0–0.009 (Additional file 7: Table S6).

Similarly, phylogenetic analysis based on the *cox*1 gene also revealed that *I. ovatus* ticks clustered into 3 separate groups (Additional file 8: Figure S2). The K2P distances within and between groups ranged between 0.002–0.010 and 0.142–0.189, respectively (Additional file 9: Table S7).

### Phylogenetic analyses and genetic distances for *Haemaphysalis* spp.

**Fig. 5:**
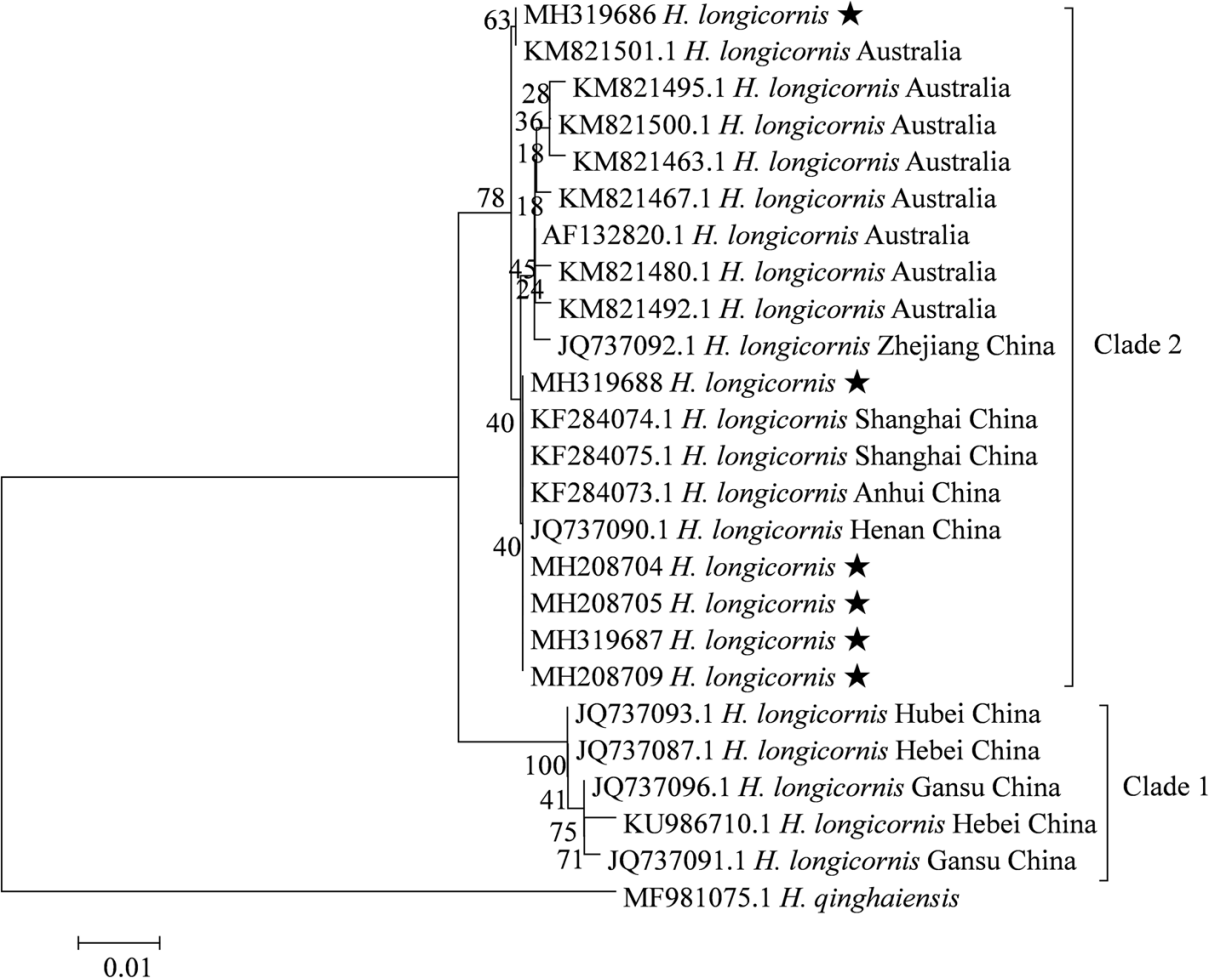
Phylogenetic tree for *H. longicornis* based on the *cox*1 gene, including sequences obtained in the present study and representative sequences from GenBank. The sequences generated in the present study are indicated with a star

According to phylogenetic analysis of the *16S* rRNA gene, all specimens of *H. longicornis* from the present study and GenBank formed a single clade (Additional file 10: Figure S3). The pairwise K2P distances of the *16S* rRNA gene ranged between 0–0.012 (Additional file 11: Table S8). However, *H. longicornis* formed two separate clades based on the *cox*1 gene (Fig. 5). Strains from Hebei, Hubei and Gansu of China clustered together, while strains from the present study and those from Australia and Zhejiang, Shanghai, Anhui, Henan of China formed another clade. The pairwise K2P distances between ticks from the two clades ranged between 0.019–0.031, while the distances between ticks from the same clade ranged between 0–0.008 (Additional file 12: Table S9).

**Fig. 6:**
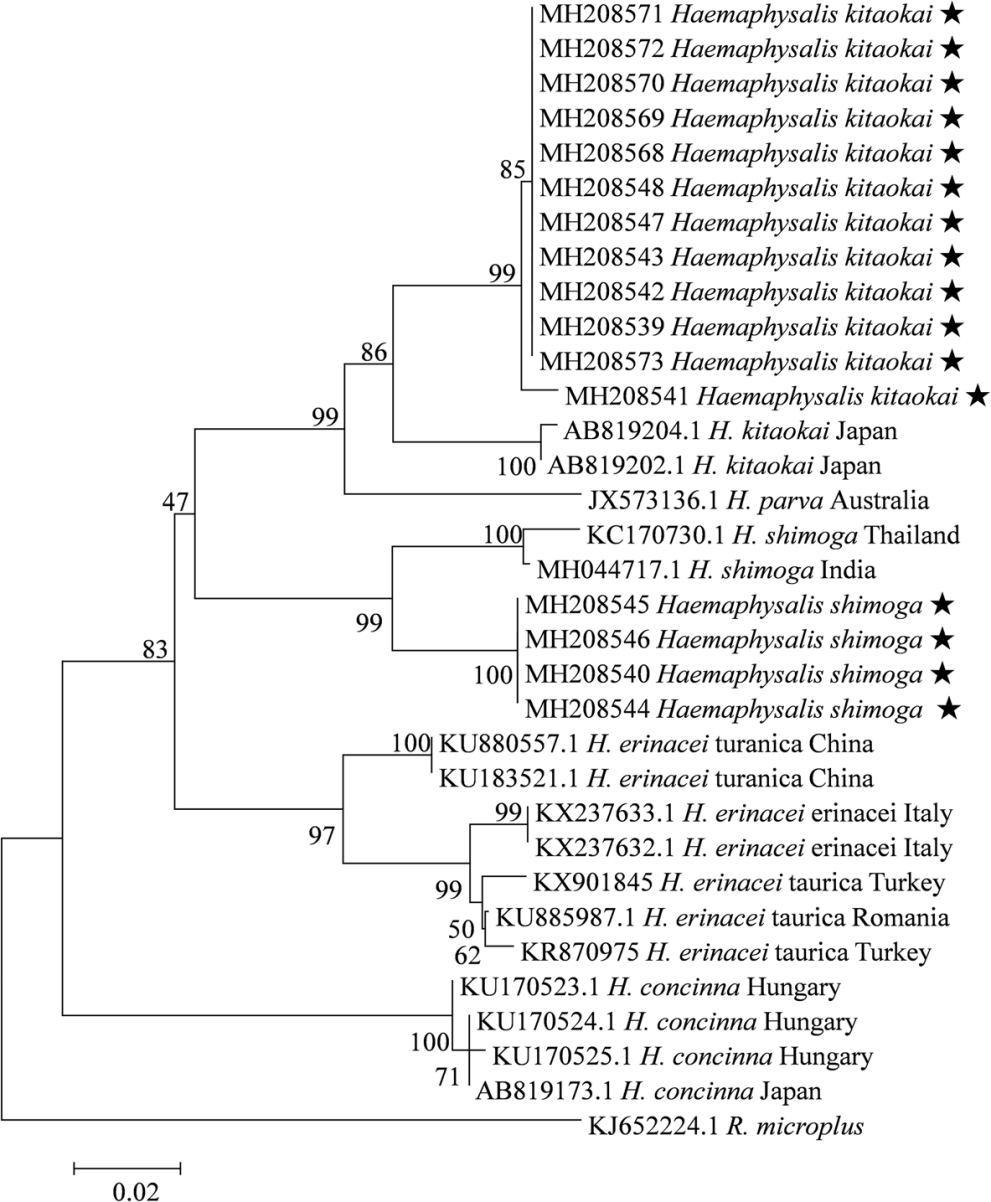
Phylogenetic tree for *H. kitaokai* and *H. shimoga* based on the *16S* rRNA gene, including sequences obtained in the present study and representative sequences of closely related tick species from GenBank. The sequences generated in the present study are indicated with a star

For *H. shimoga* ticks, 4 sequences of *16S* rRNA gene from the present study were selected for phylogenetic analysis, and 2 genetic groups were recognized: *H. shimoga* collected from India and Thailand clustered together, while all of the specimens from the present study formed a separate group (Fig. 6). Similarly, 2 genetic groups were recognized for *H. kitaokai*: all *H. kitaokai* ticks from this study formed a separate clade, while *H. kitaokai* ticks from Japan clustered together. The intergroup K2P distance ranged between 0.051–0.057 for *H. shimoga* and between 0.054–0.060 for *H. kitaokai* (Additional file 13: Table S10)*.*

## Discussion

Little is known about the genetic diversity of hard ticks in China, especially at the subspecies level [33]. In the present study, we conducted a survey on hard ticks in a China-Myanmar border county of Yunnan Province and analyzed the molecular characteristics of each tick species based on *16S* rRNA and *cox*1 gene fragments. Several tick species were identified based on morphology, including *R. microplus*, *R. haemaphysaloides*, *I. ovatus*, *H. longicornis*, *H. shimoga* and *H. kitaokai*.

*Rhipicephalus microplus* was found to be the most predominant tick species in the local area (Table 1). This species is widespread in tropical and subtropical regions and is considered to be the most important tick species infesting livestock in the world. In the present study, the *R. microplus* complex consisted of morphologically similar species difficult to distinguish. At least five taxa have been confirmed by phylogenetic analysis based on DNA barcoding, including *R. australis*, *R. annulatus* and *R. microplus* clades A-C [31, 32]. However, genetic diversity of *R. microplus* in China had not been analyzed. Our study revealed that, except for *R. australis*, all of the other four taxa were detected in China (Additional file 4: Figure S1). Ticks from the present study were clustered into *R. microplus* clade C; this clade also contains ticks from Myanmar, Bangladesh and Pakistan [34].

As far as we are aware, the genetic diversity of *R. haemaphysaloides* and *I. ovatus* had not been previously investigated. Three genetic groups were recognized based on analysis of *16S* rRNA gene, and *R. haemaphysaloides* ticks from the present study were found to belong to two of the three groups (Fig. 3). The pairwise genetic distances between ticks from different *R. haemaphysaloides* groups exceeded the recommended boundaries for describing tick species based on the *16S* rRNA gene [10]. In addition, the intergroup distances of *R. haemaphysaloides* were higher than the interspecies distances between *R. sanguineus* and *R. turanicus* (Additional file 6: Table S5). Similarly, three groups of *I. ovatus* were identified based on both genes (Fig. 4 and Additional file 8: Figure S2), and the between-group K2P distances also exceeded the recommended species boundaries for ticks (0.053 for the *16S* rRNA gene and 0.061 for the *cox*1 gene) [10, 35] (Additional file 7: Table S6 and Additional file 9: Table S7). Moreover, the genetic distances between ticks from different *I. ovatus* groups were higher than interspecies distances between *I. vespertilionis* and *I. canisuga*. The results suggest that different groups might represent distinct species or subspecies, or both *R. haemaphysaloides* and *I. ovatus* are species complexes with high genetic diversity. Unfortunately, morphological comparisons between groups were not performed in the present study. To further determine the taxonomic status of these phylogenetic groups, more studies are warranted to compare their morphological and molecular characteristics.

Few studies have investigated the molecular characteristics of *H. longicornis*. Li et al. [36] analyzed the genetic variation of *H. longicornis* from Hunan, Henan and Shandong provinces using the internal transcribed spacer (ITS) of rDNA data and found that *H. longicornis* formed a monophyletic group. Similarly, all *H. longicornis* ticks formed a single clade based on phylogenetic analysis of the *16S rRNA* gene in the present study (Additional file 10: Figure S3). However, they were clustered into two separate groups based on the *cox*1 gene (Fig. 5) and the intergroup genetic distances were higher than intragroup distances (Additional file 12: Table S9). These results suggest that *cox*1 sequences are probably more informative than *16S* rRNA sequences in revealing the intraspecies phylogenetic relationships [31]. Chen et al. [37] compared the molecular characteristics of parthenogenetic and bisexual *H. longicornis* using the *16S* rRNA gene. Their results showed that the genetic distance between the parthenogenetic and bisexual strains was closer than that between subspecies. Similarly, a phylogenetic tree cannot differentiate parthenogenetic strains from bisexual strains. For example, parthenogenetic strains from the present study and Shanghai are clustered together with bisexual strains from other regions (data not shown).

*Haemaphysalis shimoga* was first described by Trapido & Hoogstraal in 1964 [29], and *H. kitaokai* was described by Hoogstraal in 1969 [30]. Little is known about the genetic diversity of these two species. In this study, two genetic groups were recognized for both *H. shimoga* and *H. kitaokai* based on phylogenetic analysis of *16S* rRNA gene (Fig. 6). Hornok et al. [20] investigated the genetic divergence between ticks of different subspecies of *H. erinacei*. The results suggested that *H. erinacei taurica* and *H. erinacei turanica* might represent different species because the genetic divergence between them exceeded the usual divergence boundary for closely related tick species. In the present study, the intergroup genetic distances of both *H. shimoga* (0.051–0.057) and *H. kitaokai* (0.054–0.060) exceeded the distances between *H. erinacei taurica* and *H. erinacei turanica* (Additional file 13: Table S10), suggesting that the different groups of *H. shimoga* and *H. kitaokai* might represent distinct species or subspecies or that both species are species complexes with a high genetic diversity.

## Conclusions

According to the phylogenetic analysis and K2P genetic distances based on *16S* rRNA and *cox*1 genes, either new species or subspecies closely related to *R. haemaphysaloides*, *I. ovatus*, *H. shimoga* and *H. kitaokai* might exist in this China-Myanmar border county or these ticks form species complexes with highly divergent mitochondrial lineages. Morphological comparisons are warranted to further confirm the taxonomic status of these tick species.

## Additional files

Additional file 1: **Table S1.** The accession numbers for the *16S* rRNA sequences generated in the present study and the closest species sequences from the GenBank database. (XLSX 20 kb)

Additional file 2: **Table S2.** The accession numbers for the *cox*1 sequences generated in the present study and the closest species sequences from the GenBank database. (XLSX 12 kb)

Additional file 3: **Table S3.** The pairwise K2P genetic distances for *R. microplus* and other closely related *Rhipicephalus* spp. based on the *16S* rRNA gene. (XLSX 18 kb)

Additional file 4: **Figure S1.** Phylogenetic tree for *R. microplus* based on the *cox*1 gene, including sequences obtained in the present study and representative sequences of the known subspecies taxa from GenBank. Sequences obtained in this study are designated by an asterisk. (TIF 1139 kb)

Additional file 5: **Table S4.** The pairwise K2P genetic distances for *R. microplus* and other closely related *Rhipicephalus* spp. based on the *cox*1 gene. (XLSX 17 kb)

Additional file 6: **Table S5.** The pairwise K2P genetic distances for *R. haemaphysaloides* and other closely related *Rhipicephalus* spp. based on the *16S* rRNA gene. (XLSX 22 kb)

Additional file 7: **Table S6.** The pairwise K2P genetic distances for *I. ovatus* and other closely related *Ixodes* spp. based on the *16S* rRNA gene. (XLSX 19 kb)

Additional file 8: **Figure S2.** Phylogenetic tree for *I. ovatus* based on the *cox*1 gene, including sequences obtained in the present study and representative sequences from GenBank. Sequences obtained in this study are designated by an asterisk. (TIF 712 kb)

Additional file 9: **Table S7.** The pairwise K2P genetic distances for *I. ovatus* and other closely related *Ixodes* spp. based on the *cox*1 gene. (XLSX 13 kb)

Additional file 10: **Figure S3.** Phylogenetic tree for *H. longicornis* based on the *16S* rRNA gene, including sequences obtained in the present study and representative sequences from GenBank. Sequences obtained in this study are designated by an asterisk. (TIF 1672 kb)

Additional file 11: **Table S8.** The pairwise K2P genetic distances for *H. longicornis* based on the *16S* rRNA gene. (XLSX 45 kb)

Additional file 12: **Table S9.** The pairwise K2P genetic distances for *H. longicornis* based on the *cox*1 gene. (XLSX 13 kb)

Additional file 13: **Table S10.** The pairwise K2P distances of *H. kitaokai*, *H. shimoga* and other closely related species based on the *16S* rRNA gene. (XLSX 14 kb)

## Abbreviations

BLAST: Basic Local Alignment Search Tool
*cox*1: cytochrome *c* oxidase subunit 1
K2P: Kimura’s 2-parameter
NJ: neighbor-joining
