# Supplementary figures and images for: High genetic diversity in hard ticks from a China-Myanmar border county
Source: Parasit Vectors. 2018 Aug 14;11:469. doi: 10.1186/s13071-018-3048-5 (PMC6092835; doi:10.1186/s13071-018-3048-5)

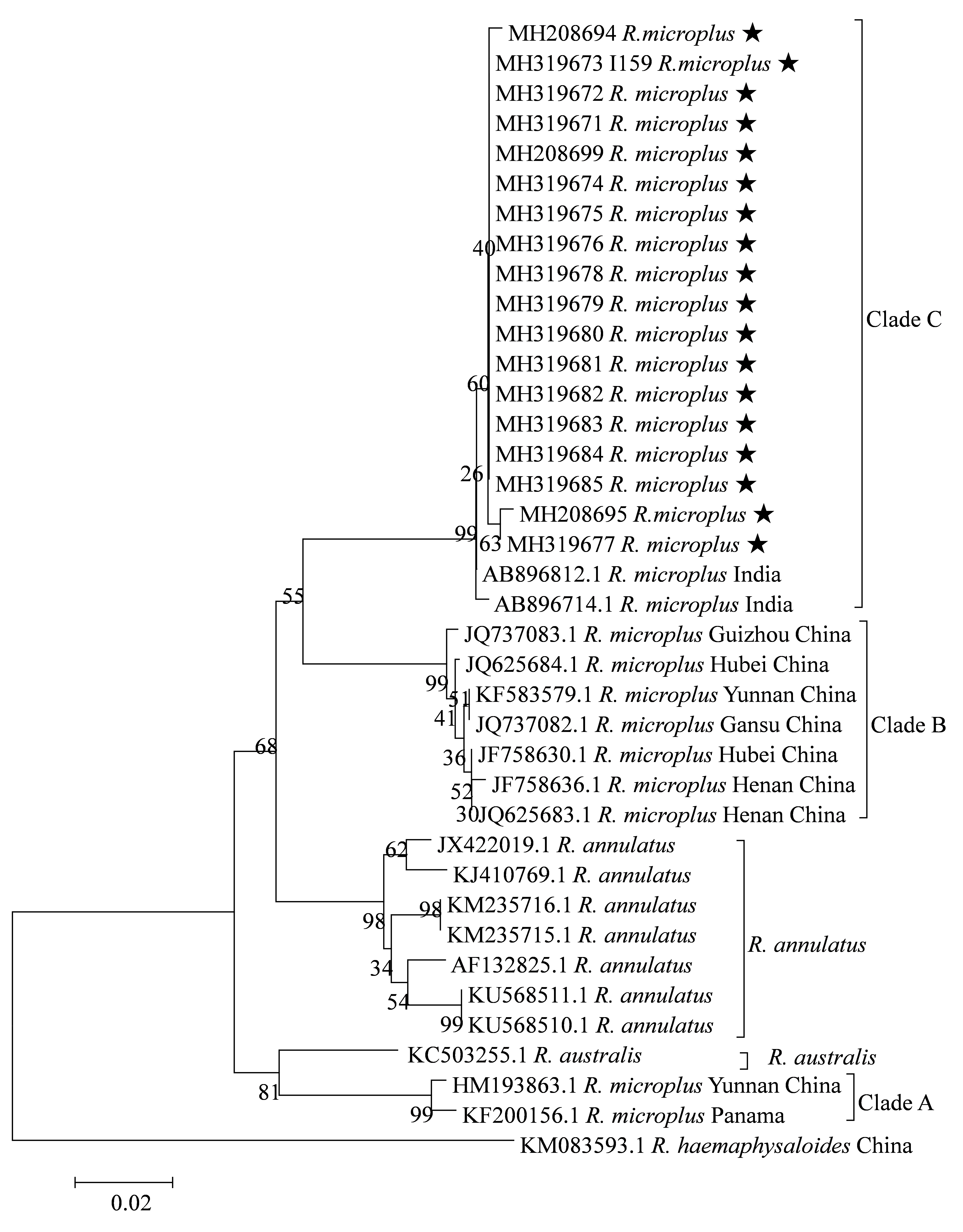

Supplement: Supplementary file 4 — Figure S1. Phylogenetic tree for R. microplus based on the cox1 gene, including sequences obtained in the present study and representative sequences of the known subspecies taxa from GenBank. Sequences obtained in this study are designated by an asterisk. (TIF 1139 kb) [file 13071_2018_3048_MOESM4_ESM.tif]

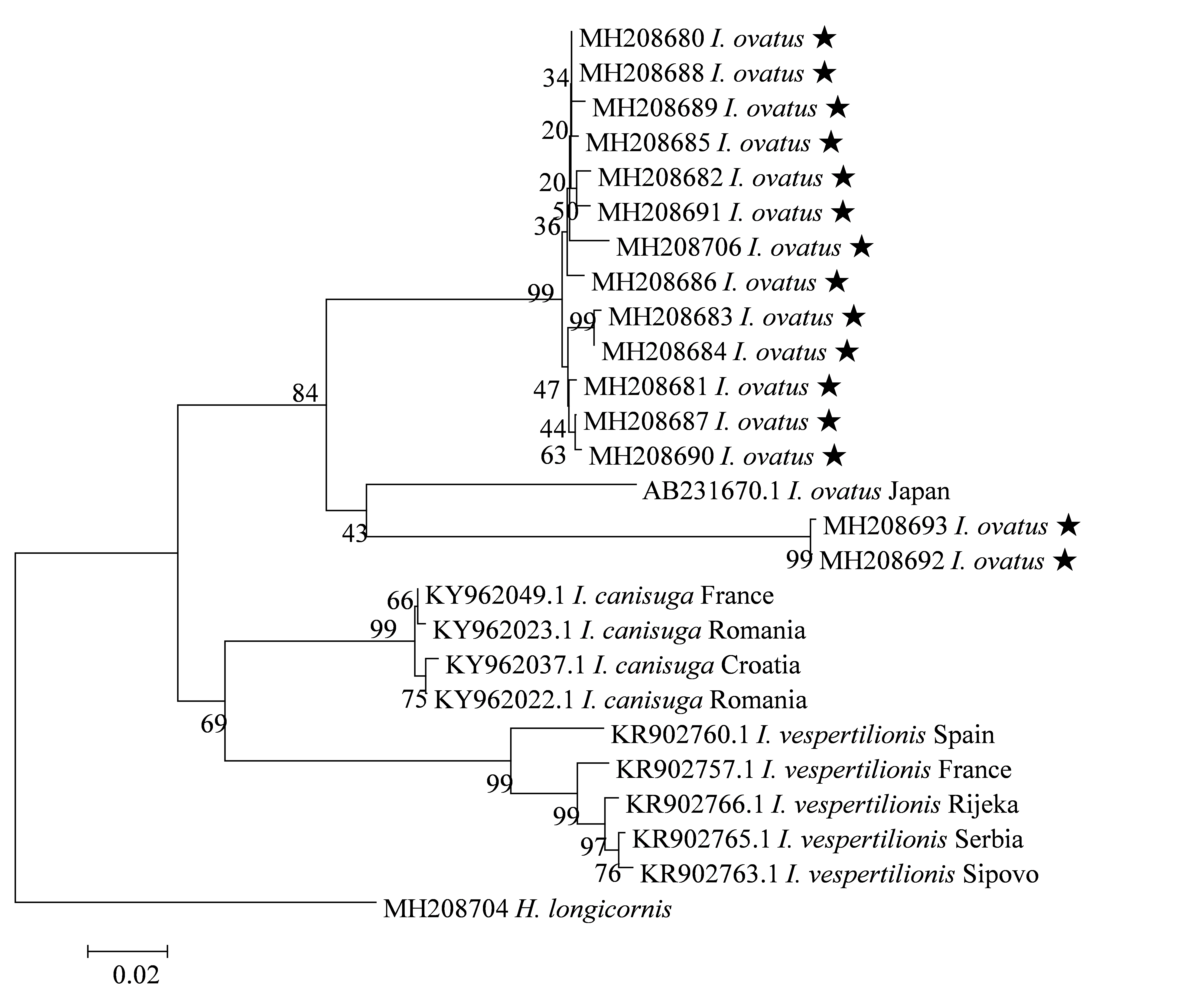

Supplement: Supplementary file 8 — Figure S2. Phylogenetic tree for I. ovatus based on the cox1 gene, including sequences obtained in the present study and representative sequences from GenBank. Sequences obtained in this study are designated by an asterisk. (TIF 712 kb) [file 13071_2018_3048_MOESM8_ESM.tif]

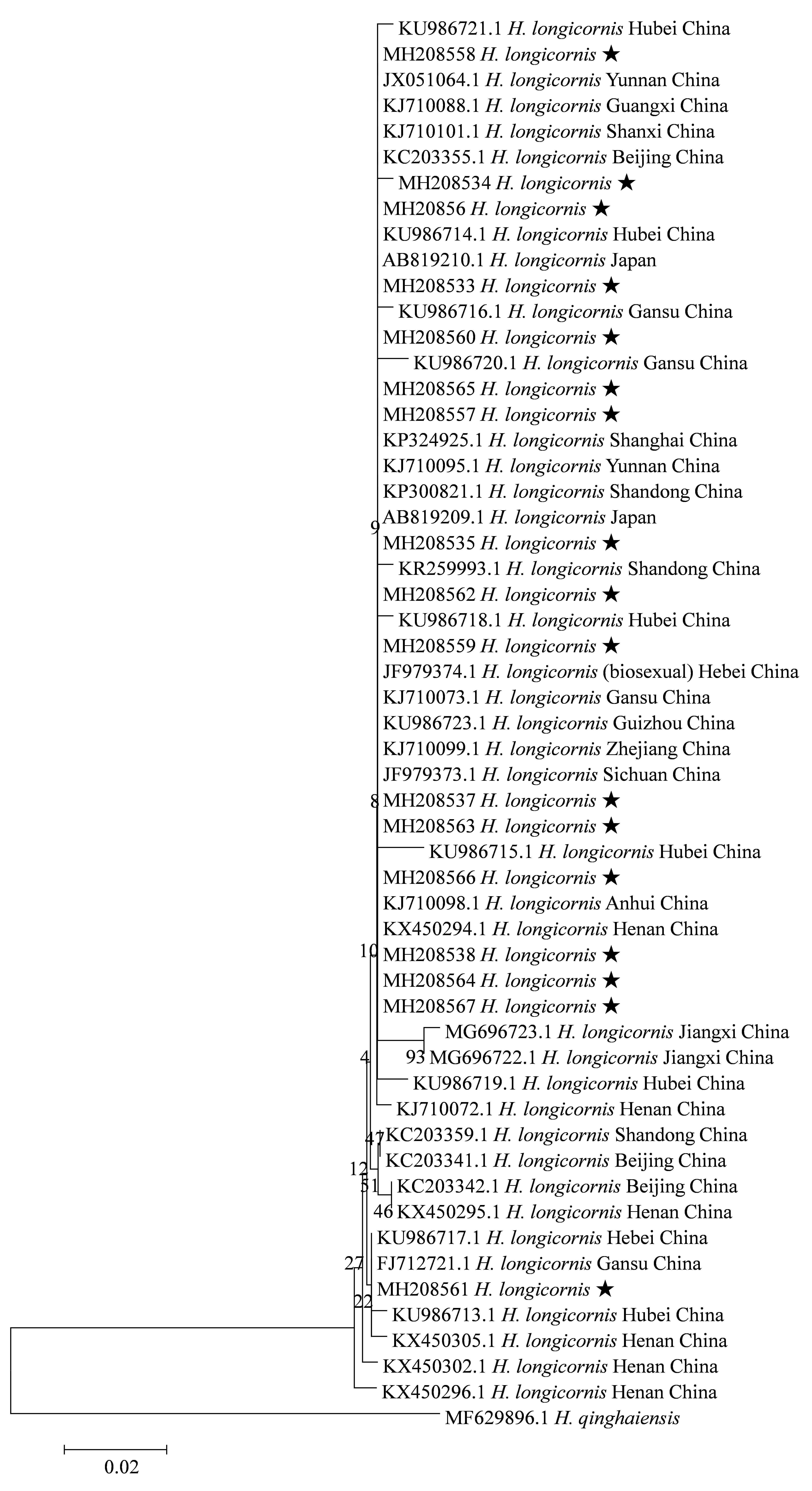

Supplement: Supplementary file 10 — Figure S3. Phylogenetic tree for H. longicornis based on the 16S rRNA gene, including sequences obtained in the present study and representative sequences from GenBank. Sequences obtained in this study are designated by an asterisk. (TIF 1672 kb) [file 13071_2018_3048_MOESM10_ESM.tif]
